# Electrodermal Activity as a Potential Diagnostic Biomarker for Alzheimer’s Disease: A Pilot Study

**DOI:** 10.3390/s26175407

**Published:** 2026-08-27

**Authors:** Sibi Pandian, Luis R. Mercado-Diaz, Jonathan Ruiz-Trivino, David Aguillon, Hugo Posada-Quintero

**Affiliations:** 1 Biomedical Engineering Department, University of Connecticut, Storrs, CT 06269, USA; sibi.pandian@uconn.edu (S.P.); luis.mercado_diaz@uconn.edu (L.R.M.-D.); 2 Grupo de Neurociencias de Antioquia, Universidad de Antioquia, Medellín 050010, Colombia; jonathan.ruiz@gna.org.co (J.R.-T.); david.aguillon@gna.org.co (D.A.)

**Keywords:** Alzheimer’s disease (AD), autonomic dysfunction, biomarkers, electrodermal activity, machine learning, signal processing

## Abstract

Alzheimer’s disease (AD), the leading cause of dementia, lacks an inexpensive and non-invasive method for early detection. Here, we investigate whether electrodermal activity (EDA), a measure of changes in electrical conductance at the skin’s surface, can offer a convenient avenue for AD screening by capturing the autonomic dysfunction associated with the disease’s pathology. As few studies have comprehensively examined EDA’s utility as a standalone biomarker for AD, we conducted a pilot study with 10 cognitively healthy controls and 20 patients with AD, evaluating EDA recordings collected during an orthostatic test and a Montreal Cognitive Assessment (MoCA). The AD group included both carriers from Colombian kindreds affected by autosomal-dominant AD and patients with sporadic late-onset AD. We compared EDA features between groups using statistical analysis and tested whether machine learning models could separate the two groups using these features. During the MoCA, phasic EDA measures, specifically the number of skin conductance responses (NSSCR) and mean time-variant sympathetic tone (TVSymp), were significantly lower in the AD group than in controls (NSSCR: p=0.0083, Cohen’s d=−1.07; mean TVSymp: p=0.0166, d=−0.96), suggesting a blunted sympathetic response to cognitive stress in AD. No significant between-group differences were observed during the orthostatic test (all p≥0.60). A random forest classifier trained on MoCA-derived EDA features achieved a balanced accuracy of 78% (95% CI 60 to 93%; 75% sensitivity, 80% specificity, AUROC 0.75), with phasic features contributing most to performance. Performance did not differ significantly from that of the support vector machine or logistic regression models. These results provide preliminary, hypothesis-generating evidence for the utility of EDA in screening for AD. The small sample size, unequal group allocation, sex imbalance, and substantial mean age disparity between groups (≈23.5 years) limit the strength of these conclusions and necessitate replication in larger, age- and sex-matched cohorts that include prodromal stages.

## 1. Introduction

Dementia is the world’s seventh leading cause of death, with Alzheimer’s disease (AD) being the most prevalent cause, accounting for 60 to 70% of reported dementia cases [1]. It is estimated that by 2050, the number of dementia patients will expand from 50 to 152 million, costing the world trillions of dollars in healthcare and caretaking expenditure [2]. While there currently exists no medical treatment capable of reversing the pathological and cognitive changes associated with AD, the disease’s symptoms appear in progressive stages that eventually terminate in dementia [3]. Given the progressive nature of AD, an emerging focus of recent literature has been to detect the disease in its early stages [3,4]. In the interest of locating a non-invasive method of AD detection, this study investigates whether electrodermal activity (EDA), a measure of the changes in electrical conductance at the skin’s surface due to sweat gland activity, can be used to identify AD.

Early AD diagnosis may require the classification of underlying neurophysiological events related to the pathological progression of the disease, rather than a diagnosis exclusively through clinical symptoms [5,6]. For example, mild cognitive impairment (MCI) broadly represents a state of cognitive decline abnormal for a given age, yet does not meet the clinical criteria for dementia; however, individuals with the amnestic variant of MCI are at high risk of developing dementia due to AD [7,8], indicating that the onset of AD pathology may occur at the MCI stage or earlier.

A promising approach for early AD detection is the development of biomarkers that can reflect these pathological changes. Biomarkers based on PET, cerebrospinal fluid assays, and more recently plasma-based assays have been developed to detect abnormalities in amyloid and tau protein concentrations, which function as early pathological markers of the disease [9,10]. Less commonly, MRI has also been used to identify biomarkers based on neurodegeneration and altered brain connectivity [10]. Early detection through biomarkers is particularly valuable because it enables individuals to plan for their condition [3], improves clinical outcomes [11], and may increase the effectiveness of emerging therapies before substantial neurodegeneration has occurred [12]. In spite of the considerable success of the aforementioned techniques in identifying AD, their widespread employment is limited by the costly and invasive nature of these methods.

An alternate route to detecting AD lies in its well-reported comorbidity with autonomic dysfunction [13,14]. The pathology tying autonomic dysfunction with AD is not fully understood, with studies potentially attributing this link to structural changes in the central autonomic network or to failures in the cholinergic system, which plays a critical role in both cognitive function and parasympathetic regulation [14].

Regardless, the symptoms of autonomic dysfunction may manifest in AD subjects even prior to the onset of dementia [13], with [15] finding significant differences using heart rate variability (HRV) analysis in populations with and without MCI, demonstrating autonomic dysfunction’s potential as a biomarker for AD. Further support is found in a 20-year longitudinal study reporting a 20% incidence of orthostatic hypotension (OH) in dementia populations [16,17], and in fMRI studies conducted in AD populations which reveal impaired autonomic responses [18,19].

Specifically, studies highlight autonomic dysfunction in the form of impaired sympathovagal balance and sympathetic nervous system dominance [20,21]. Therefore, as a signal that reflects the predominantly sympathetic innervation of sweat gland activity [22], EDA may hold significant promise in detecting AD.

Although HRV is the most widely used peripheral index of autonomic function in AD research, EDA offers several complementary advantages that motivate its use here. First, EDA is a comparatively selective probe of sympathetic outflow, because eccrine sweat glands receive essentially exclusive sympathetic cholinergic innervation, so the signal is not the resultant of two opposing branches of the autonomic nervous system. Most HRV indices, by contrast, reflect the combined and often confounded influence of sympathetic and parasympathetic activity [22,23]. Second, HRV is strongly modulated by respiration: respiratory sinus arrhythmia contaminates the high-frequency band, and low-frequency HRV requires several minutes of stationary recording to be estimated reliably [24]. EDA is far less susceptible to respiratory artifacts, and phasic EDA features such as the skin conductance response count can be extracted from shorter and less strictly controlled recordings. Third, EDA acquisition is non-invasive and inexpensive, typically obtained using two electrodes placed at the fingertips, where a high concentration of sweat glands is present, and it is already routinely embedded in consumer-grade wrist-worn devices, which makes it attractive for the remote and longitudinal screening applications we envisage. These advantages come at a cost. EDA is more sensitive than HRV to skin properties, ambient temperature and humidity, movement, and electrode-to-skin interface effects, and these factors must be controlled at acquisition time (Section 2.3).

Although challenging to analyze due to its nonlinear and non-stationary properties, recent advancements in EDA processing methodologies have amplified the signal’s usage and reliability [22]. These include the development of sophisticated algorithms to separate the signal’s phasic and tonic components [25,26] and the development of ‘TVSymp’, a highly sensitive EDA feature for sympathetic activation over time [23]. Studies have been successful in utilizing EDA for diverse purposes such as assessing cognitive fatigue in military personnel [27], analyzing emotional states [28], and identifying neurological conditions such as autism [29]. Moreover, several studies have found promising results using continuous EDA measurement to assist in caring for populations with dementia, suggesting that it may be well-suited for wearable device-based symptom tracking and home-based care settings for patients with AD [30,31,32,33].

Despite this activity, the existing literature does not establish whether EDA alone carries information about AD status, and the present study is positioned to fill three specific gaps. First, the dementia-oriented EDA literature has used the signal almost exclusively as a monitoring tool in patients already diagnosed, to flag agitation, problematic behavior, or stress episodes in care settings [30,31,32,33], rather than as a discriminative measure evaluated against a control group. Second, the one study that has examined EDA in relation to AD status [34] embedded EDA inside a multimodal, emotion-elicitation feature set alongside other physiological and behavioral modalities, so the independent contribution of EDA could not be isolated. Here, all features are derived from a single EDA channel, and no other modality enters the models. Third, prior work has relied on conventional tonic and amplitude-based EDA descriptors, whereas we evaluate spectral and time-frequency indices of sympathetic control (TVSymp and EDASympn) [23] that have not previously been applied to an AD population, and we do so during two standard clinical maneuvers, a cognitive challenge and an orthostatic challenge, that are already administered in routine checkups and are directly transferable to telehealth settings. We therefore present a pilot study investigating the standalone utility of EDA features to distinguish between AD and control populations, analyzing EDA recordings acquired during the administration of cognitive and orthostatic tests in a clinical setting.

We emphasize at the outset that the design of this study is a case–control comparison between individuals with an established clinical diagnosis of AD at the dementia stage and cognitively healthy individuals. It therefore cannot speak to diagnostic accuracy in the setting where a screening test would actually be deployed, namely the separation of prodromal or early-stage disease from normal aging. No participants with MCI or preclinical AD were included, the two groups differ markedly in age and sex composition, and the sample is small. Accordingly, we frame our results throughout as preliminary evidence that EDA is sensitive to AD status under cognitive challenge, which is a necessary but far from sufficient condition for diagnostic utility, and as motivation for the appropriately designed studies that would be required to establish it.

## 2. Materials and Methods

### 2.1. Participants

Participants were recruited during monthly clinical checkups at the Grupo de Neurociencias de Antioquia (GNA), Universidad de Antioquia, Medellín, Colombia, and all provided written informed consent. EDA was recorded from 30 participants: 20 with a clinical diagnosis of AD at the dementia stage, established according to the NIA-AA clinical criteria [35] by the attending neurologist, and 10 cognitively healthy controls. Functional severity was staged with the Functional Assessment Staging Tool (FAST) [36].

The recruiting center maintains two parallel clinical cohorts, and participants in the present study were drawn from both. The first is a longstanding registry of Colombian kindreds affected by autosomal-dominant AD, in which affected individuals develop dementia in their late forties and early fifties [37,38]; unaffected relatives from the same families serve as the natural comparison group and are correspondingly young. The second is a general memory clinic serving patients with sporadic late-onset AD. Of the 20 participants in the AD group, 6 were from the autosomal-dominant kindreds and 14 were recruited through the sporadic late-onset arm; of the 10 controls, 8 were unaffected members of the kindreds and 2 were community volunteers recruited alongside the sporadic arm. This composition is important for the interpretation of our results and is examined in Section 3.3 and Section 4.3. Controls were free of any diagnosis of dementia, MCI, or other neurological disorder, and none was taking centrally acting or cardiovascular medication.

Two points about the composition of the AD group are stated explicitly. First, 19 of the 20 patients were staged at the dementia level of severity, while 1 participant recruited through the sporadic arm was staged as amnestic mild cognitive impairment at the time of assessment, with a MoCA score of 25 and a FAST stage of 1. This participant was retained because the study was designed around the AD continuum rather than around a single severity stratum, but the patient group is therefore more accurately described as spanning the symptomatic AD spectrum than as a pure dementia sample. We report a sensitivity analysis excluding this participant in Section 3.3, and we note the implications of our findings in Section 4.3. Second, one further participant from the autosomal-dominant kindreds completed the assessment but was excluded from all analyses because of a fault in data collection that rendered the electrodermal recording unusable. The exclusion was made on this objective criterion, before any group comparison was performed, and applies equally to the MoCA and orthostatic test recordings.

This is an exploratory pilot study, and the sample size was not derived from an a priori power calculation. Both groups were convenience samples comprising the eligible participants who attended the clinic during the recruitment window, and no eligible participant was excluded in order to meet a sample-size target. The 20 to 10 allocation, which reverses the asymmetry usual in case–control studies, reflects the composition of that clinic population: patients attending scheduled checkups for an established diagnosis were more readily available than cognitively healthy volunteers willing to undertake the full assessment protocol. The same constraint accounts for the sex imbalance reported in Table 1, since the participating families and clinic attendees were not sampled with sex balance in mind.

The consequences of this design for statistical power, for the reliability of the classification models, and for the generalizability of the findings are examined in Section 4.3, where we also report the sample sizes that an adequately powered replication would require [39,40].

Medication status was ascertained from the clinical history recorded at the study visit and is summarized in Table 1. No control participant was taking a cholinesterase inhibitor, an antihypertensive, or a beta-blocker. Within the AD group, 6 of the 20 participants (30%) were receiving a cholinesterase inhibitor: rivastigmine in 3, galantamine in 2, and donepezil in 1. Because cholinesterase inhibitors act on the same cholinergic system that mediates sudomotor secretion, and because their net effect on autonomic balance is dose- and duration-dependent [41], we treated cholinergic therapy as a potential modifier of the EDA measures and performed a sensitivity analysis excluding treated participants (Section 2.5).

### 2.2. Test Protocols

**Figure 1:**
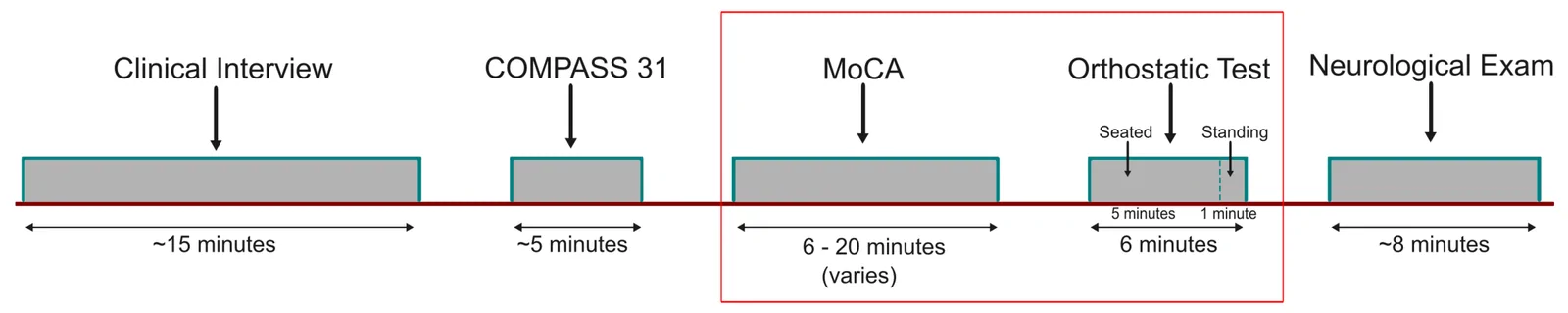
Structure of the assessment session. Each participant completed, in this order, a clinical interview, the COMPASS-31 autonomic symptom questionnaire, the Montreal Cognitive Assessment, the orthostatic test, and a neurological examination. Approximate durations are given beneath each block. Electrodermal activity was recorded continuously from the start of the session. The two segments analyzed in the present study are outlined in red: the Montreal Cognitive Assessment, which has no fixed time limit and took between 6 and 20 min, and the orthostatic test, comprising 5 min seated followed by 1 min standing.

As part of the checkup, subjects participated in a fixed sequence of assessments, shown in Figure 1: a clinical interview of approximately 15 min, the COMPASS-31 autonomic symptom questionnaire [42] of approximately 5 min, a Montreal Cognitive Assessment (MoCA) [43], an orthostatic test, and a neurological examination of approximately 8 min. Electrodermal activity was recorded continuously across the whole session, and the present analysis uses the two segments outlined in Figure 1, namely the MoCA and the orthostatic test. Note that the MoCA preceded the orthostatic test, so the cognitive challenge was always administered before the postural one. For the MoCA, subjects were provided a 30-point questionnaire designed to assess whether the subject demonstrates signs of MCI, testing their short-term memory, executive functioning, and other cognitive capacities. Consistent with standard MoCA administration, no time limit was imposed on the assessment as a whole, and the EDA recording spanned the interval from the first to the last administered item. Administration times were logged for every participant and are reported in Table 1; the implications of the variable duration for feature computation are addressed in Section 2.4.

For the orthostatic test, subjects were instructed to remain seated for 300 s, and to stand up for 60 s afterwards. Blood pressure measurements were taken at the start of the test and immediately after the subject stood up, to check for a sharp decline in blood pressure, defined as a systolic drop of ≥20 mmHg or a diastolic drop of ≥10 mmHg. The 60 s standing interval was fixed by the clinical protocol already in use at the recruiting center, where the maneuver is performed to screen for symptomatic orthostatic hypotension. In that context, the relevant blood-pressure fall occurs within the first minute of standing, and the duration is deliberately kept short because prolonged standing is poorly tolerated by patients with moderate to severe dementia and carries a fall risk. The EDA recordings analyzed here were therefore acquired opportunistically within a pre-existing clinical protocol rather than under a protocol designed for autonomic signal analysis.

### 2.3. EDA Acquisition and Recording Conditions

EDA was acquired with a commercial Shimmer 3 GSR+ unit at a sampling rate of 46 Hz, using the exosomatic constant-voltage method. Acquisition conditions are reported below in accordance with the publication recommendations for electrodermal measurements [44].

Electrodes. We used the reusable dry Ag/AgCl finger contacts supplied with the Shimmer GSR+ kit, applied to the volar surface of the distal phalanges of the index and middle fingers. No electrolyte gel or paste was used at any point. This choice removes one common source of variability, since a hypertonic conductive medium can raise measured conductance and its effect depends on how long the gel has been in contact with the skin, but it introduces another, because dry contacts have a higher and more variable electrode-to-skin impedance than wet electrodes and require a longer interval to stabilize. The stabilization procedure described below was adopted for that reason. A single electrode pair was used for all participants rather than a fresh pair per participant. The pair was cleaned between participants following the manufacturer’s instructions for the GSR+ kit, and participants washed their hands with unscented soap and water and dried them before the electrodes were applied. No alcohol, abrasive, or antiperspirant preparation was applied to the recording site, since these alter stratum corneum hydration and the electrode-to-skin interface.

Electrode stabilization. Electrodes were applied at the very beginning of the session, before the clinical interview, and the recording ran continuously from that point onward. Because the clinical interview and the COMPASS-31 questionnaire precede the first analyzed segment (Figure 1), approximately 20 min elapsed between electrode application and the start of the MoCA recording, and approximately 30 min before the seated phase of the orthostatic test. This is well beyond the stabilization interval recommended for dry-contact recordings, so the hydration of the stratum corneum at the contact site and the skin conductance level had settled long before any analyzed feature was computed. The 5 min seated phase of the orthostatic test therefore functioned purely as a postural baseline rather than as an electrode settling period.

Posture, movement and speech. During both protocols, the recording hand rested, palm upward and fingers relaxed, on an armrest or on the participant’s thigh, and participants were instructed to keep that hand still and to avoid gripping or fidgeting movements. The non-recording hand was used for any manual component of the tasks. During the orthostatic test, participants were additionally instructed not to speak; when standing, they were asked to remain still with the recording arm relaxed at their side. The MoCA, however, is an interactive verbal assessment: participants necessarily speak while responding, and the examiner speaks while administering the items. Speech and vocal effort are themselves elicitors of skin conductance responses, so this is an inherent feature of the paradigm rather than something that could be eliminated. Factors mitigating the concern of motion artifacts are considered in Section 4.3.

Environment. All recordings were conducted indoors with the participant seated in a chair. Because ambient temperature and relative humidity systematically affect tonic level and response amplitude [45,46], all sessions were conducted under fixed climate-controlled conditions to ensure both factors remained constant across participants and groups.

We did not, however, log temperature and humidity continuously during individual sessions, and we therefore report the environment as controlled rather than as instrumented; this is noted again in Section 4.3. Sessions were conducted with the door closed and without background music or conversation from third parties, in order to limit extraneous auditory stimuli, which are known to evoke skin conductance responses and to bias EDA measurements [47].

### 2.4. Data Preprocessing and Feature Extraction

We first applied a one-second window median filter to the EDA signals to mitigate artifacts arising from movement or electrode displacements. Subsequently, a 1 Hz FIR low-pass filter was applied, based on evidence that the spectral content of EDA signals lies below 0.5 Hz [48]. We then downsampled the signals from 46 Hz to 8 Hz to improve data processing efficiency, and extracted the tonic and phasic components using the sparsEDA algorithm [25].

To obtain the time-variant sympathetic tone, TVSymp, and the time-invariant sympathetic index, EDASympn, we further downsampled the signal to 2 Hz and applied a 4th-order Butterworth bandpass filter to preserve the frequency range considered reflective of sympathetic arousal, 0.01 to 0.24 Hz [23].

High-amplitude motion and noise artifacts were detected with the EDAQA tool [49]. We set this tool to identify samples where values exceeded 100 µS or fell below 0 µS, as well as samples where the signal changed very rapidly, that is, with a slope greater than 0.5 µS/s. When a sample violated either of these rules, the sample and the two samples immediately preceding and following it were determined to be invalid. During feature computation for a given segment of the EDA signal, the invalid samples were removed from the segment. The signals were visually inspected to ensure that removing the invalid samples did not result in any sharp artifacts. Only a small proportion of samples were identified as invalid and removed, averaging 0.78% (SD = 1.53%) for the MoCA recordings and 0.51% (SD = 0.83%) for the orthostatic test recordings. This low rejection rate is consistent with the procedural movement controls described in Section 2.3.

We extracted the following EDA features from the signals: mean skin conductance level (SCL), number of skin-conducted responses (NSSCR), mean TVSymp, and EDASympn. TVSymp was calculated with the VFCDM approach as described in [23], and EDASympn was calculated as the sum of spectral amplitudes in the 0.01 to 0.24 Hz range normalized to the total power of the signal, with spectral amplitudes found using Welch’s method with 32-sample, 50% overlap Hamming windows.

All four features are, by construction, invariant to recording length rather than cumulative over it. NSSCR is expressed as a rate, in responses per minute; SCL and mean TVSymp are temporal averages of their respective components; and EDASympn is a ratio of spectral power in the sympathetic band to total power. Since no feature is a raw count or a raw sum, they are not mechanically inflated by a longer MoCA administration, and thus no further normalization by duration is applied.

This preprocessing and feature extraction protocol was carried out identically for the EDA signals from the orthostatic test and the MoCA periods. We divided the orthostatic test signals into segments based on orthostatic position, that is, whether the subject was seated or standing, before feature calculation.

### 2.5. Statistical Analysis and Machine Learning

The normality of each EDA feature was independently assessed across subjects using the Kolmogorov–Smirnov test.

For both the orthostatic test and MoCA, between-group comparisons of EDA features were performed using unpaired *t*-tests, with the Wilcoxon rank-sum test applied when normality assumptions were violated. In all cases, we used an initial significance threshold of p<0.05. To account for multiple comparisons, a Bonferroni correction was applied for the four feature-wise comparisons within each test, providing an adjusted significance threshold of p<0.0125 (0.05/4). All statistical analyses were performed using MATLAB R2026a (The MathWorks, Inc., Natick, MA, USA) with the Statistics and Machine Learning Toolbox, except for the power calculations, which used the *statsmodels* 0.14.6 library in Python 3.14 [50].

#### 2.5.1. Covariate-Adjusted and Stratified Analyses

The two groups differ substantially in both age and sex composition (Table 1), and both variables are known to influence EDA independently of disease status [51,52]. To assess the extent to which the observed group differences survive adjustment for these variables, we refitted the MoCA comparisons as multivariable linear models of the form

$$\mathrm{feature}∼\beta_{0}+\beta_{1}\;\mathrm{group}+\beta_{2}\;\mathrm{age}+\beta_{3}\;\mathrm{sex}+\varepsilon ,$$

estimated by ordinary least squares, and report the partial effect of group ($\beta_{1}$), its *p*-value, and the partial $\eta^{2}$.

We stress that these models should be read as sensitivity analyses and not as de-confounded estimates of an AD effect. Group and age are strongly collinear in this cohort, and adjustment for a covariate that is nearly a proxy for group membership does not recover an unbiased group effect. It merely quantifies how much of the association is statistically attributable to group once the shared variance is removed, with wide standard errors. We therefore additionally report the point-biserial correlation between group and age, the variance inflation factor for the group term, and the proportion of the age distributions that overlaps.

We complemented the regression models with three designs that remove a confounder by construction rather than by adjustment.

- Etiology-restricted analysis. The heterogeneous composition of our AD group creates an opportunity that a homogeneous cohort would not offer. Participants from the autosomal-dominant kindreds develop dementia in their late forties and early fifties [37,38] and are therefore close in age to our controls, whereas the sporadic late-onset participants are roughly three decades older. We repeated the primary MoCA comparisons using only the autosomal-dominant AD participants against the full control group, a contrast in which the mean age difference falls from 23.5 years to 6.4 years.
- Sex-restricted analysis. We repeated the primary comparisons using only the female participants, of whom there are 9 in each group.
- Medication-restricted analysis. We conducted an analysis excluding AD participants receiving cholinesterase inhibitor therapy [41].

As stated, these are reported as sensitivity analyses, with the unadjusted results retained as the primary outcome.

#### 2.5.2. Classification Models

Machine learning models were applied to test whether we could separate AD and control subjects using all four EDA features, independently for the orthostatic test (using the difference in features between the sitting and standing phases) and the MoCA. Three types of models were implemented in MATLAB using default functions: linear support vector machines (*fitcsvm*, linear kernel), logistic regression (*fitclinear*), and random forests (*TreeBagger*). Leave-one-subject-out cross-validation was employed to minimize overfitting and to evaluate how well the model generalizes to unseen subjects. For each split, we first used the K-nearest-neighbor-based Synthetic Minority Oversampling Technique (SMOTE) [53] to balance the training set. SMOTE was applied strictly inside the training fold of each split and never to the held-out subject.

Then, for all models except random forest, the data were standardized to have zero mean and unit variance. To optimize the hyperparameters of the models, we used grid-search cross-validation, further dividing the training set into five folds. We then selected the hyperparameters that yielded the highest balanced accuracy score, averaged over all five folds. The balanced accuracy score is defined as follows:

$$\mathrm{Balanced}\;\mathrm{Accuracy}=\frac{\mathrm{Sensitivity}+\mathrm{Specificity}}{2}$$

For the support vector machine and logistic regression models, the hyperparameters were the *BoxConstraint* and *Lambda* parameters, selected from 0.01, 0.1, 1, 10, 100, and 1000. For the random forest model, the hyperparameters were the maximum tree depth (choices were 3, 4, and 5) and the number of trees (50, 100, and 200).

Model performance was quantified by sensitivity, specificity, balanced accuracy, and the area under the receiver operating characteristic curve (AUROC), each reported with a 95% confidence interval. Confidence intervals for sensitivity and specificity were computed as Wilson score intervals [54]; the interval for balanced accuracy was obtained by a stratified subject-level bootstrap of the leave-one-subject-out predictions with 10,000 resamples [55]; and the interval for the AUROC was obtained by the method of Hanley and McNeil [56]. Because the three classifiers were evaluated on exactly the same held-out subjects, their predictions are paired and were compared using McNemar’s exact test on the subject-level predictions [57]. We report these comparisons explicitly rather than ranking the models by point estimates alone, since with n=30, a difference of one correctly classified subject shifts balanced accuracy by roughly 2.5 percentage points.

Feature contributions were assessed by permutation importance based on balanced accuracy. Given the sample size, permutation importance estimates are expected to be unstable, and we therefore report the mean performance drop over repeated permutations of each feature and interpret only the ordering of the largest contributors, not the precise magnitudes [58,59].

## 3. Results

### 3.1. Participant Characteristics

The two groups differed as expected in cognitive status, with a mean MoCA score of 24.6 ± 3.3 in controls and 9.2 ± 6.2 in the AD group, and also differed substantially in age (44.1 ± 6.91 versus 67.6 ± 16.22 years) and in sex composition (9 of 10 female participants in the control group versus 9 of 20 female participants in the AD group; Table 1). The AD group was heterogeneous in etiology, comprising 6 participants from the autosomal-dominant kindreds, whose mean age was 50.5 years, and 14 participants recruited through the sporadic late-onset arm, whose mean age was 74.9 years.

Orthostatic symptom burden was assessed with the COMPASS-31 autonomic symptom questionnaire [42], administered earlier in the same session (Figure 1). A total of 9 of the 20 AD participants (45%) and 4 of the 10 controls (40%) reported orthostatic symptoms, a difference that is not statistically significant (p=1.00). However, applying the pre-specified blood-pressure criterion to the single pair of seated and standing readings obtained during the orthostatic test itself, one control participant and no AD participant met the definition. We report both figures because the discrepancy is informative, and we note that they measure different things: COMPASS-31 quantifies self-reported symptom burden, whereas the blood-pressure criterion is a haemodynamic measurement made at one instant.

### 3.2. Between-Group Comparison of EDA Features

The statistical analysis tested whether we could distinguish physiological responses between the AD and control groups using features derived from EDA. Table 2 summarizes the results of the four EDA features derived during the orthostatic test and the MoCA.

**Figure 2:**
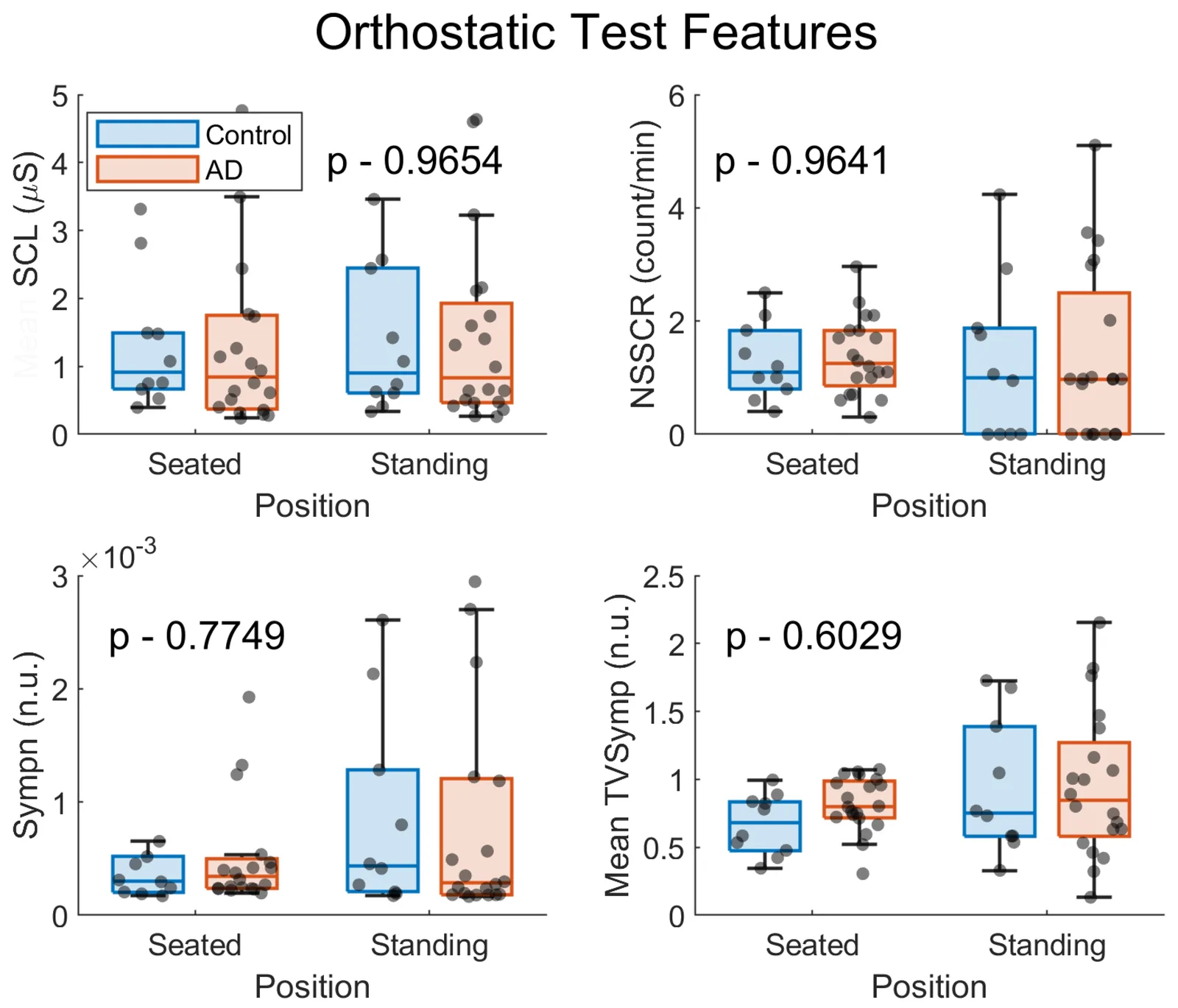
Paired box plots of the four EDA features during the seated and standing segments of the orthostatic test, for the control (blue) and AD (orange) groups. Individual participants are overlaid as points. The *p*-value annotated in each panel is for the between-group comparison of the standing-minus-sitting change in that feature. SCL: skin conductance level; NSSCR: number of skin conductance responses; Sympn: normalized time-invariant EDA sympathetic index; TVSymp: time-variant sympathetic tone; n.u.: normalized units.

During the orthostatic test, the EDA features showed no significant differences between the AD and control groups (SCL: p=0.9654; NSSCR: p=0.9641; EDASympn: p=0.7749; mean TVSymp: p=0.6029), as shown in the paired box plots in Figure 2.

**Figure 3:**
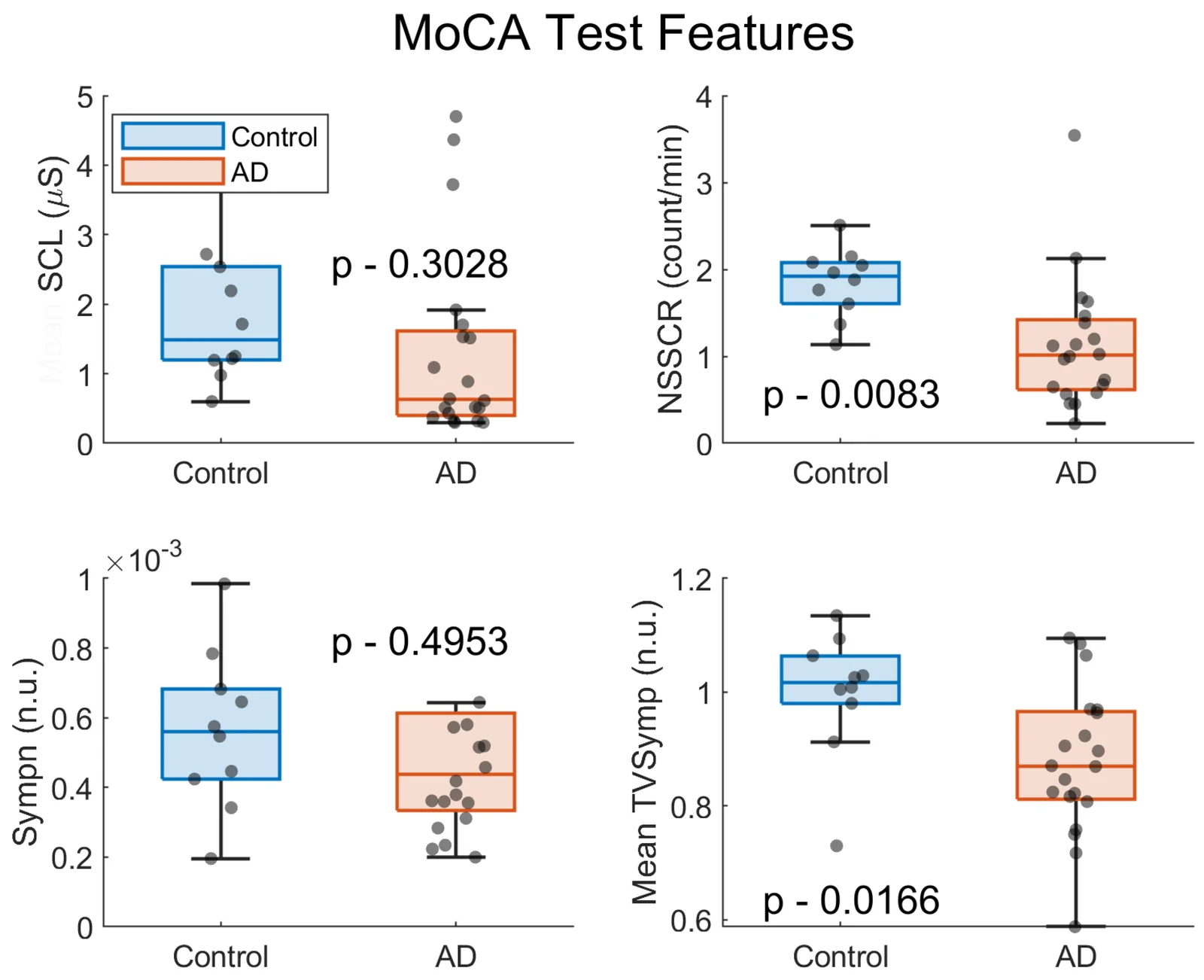
Box plots of the four EDA features recorded during the Montreal Cognitive Assessment, for the control (blue) and AD (orange) groups. Individual participants are overlaid as points. The *p*-value annotated in each panel is for the between-group comparison of that feature. SCL: skin conductance level; NSSCR: number of skin conductance responses; Sympn: normalized time-invariant EDA sympathetic index; TVSymp: time-variant sympathetic tone; n.u.: normalized units.

The results for the MoCA are summarized in Figure 3. Here, NSSCR and mean TVSymp showed significant differences between the AD and control groups (NSSCR: t(28)=−2.84, p=0.0083, Cohen’s d=−1.07; mean TVSymp: t(28)=−2.55, p=0.0166, d=−0.96), with AD subjects exhibiting lower values. SCL and EDASympn did not differ significantly between groups (p=0.3028 and p=0.4953, respectively). After Bonferroni correction for multiple comparisons, only the difference in NSSCR remained significant, though both features showed consistent changes and similar effect sizes.

### 3.3. Covariate-Adjusted and Stratified Analyses

Age and group membership were strongly associated in this cohort (point-biserial r=0.63, p<0.001), and the two age distributions overlapped only over the range of 46 to 63 years, a window that contains 3 of the 10 controls and 9 of the 20 AD participants. Adjustment for age therefore attempts to separate two variables that this sample barely distinguishes, and the results below should be interpreted in that light. Table 3 reports the group effect for the two MoCA features that were significant in the unadjusted analysis, before and after adjustment for age and sex.

The pattern in Table 3 is similar for both features. Adjusting for sex leaves the group effect essentially unchanged: the partial regression coefficient for NSSCR moves from −0.720 to −0.858 and remains significant, and the coefficient for mean TVSymp is stable at −0.121 and −0.123. Adjusting for age removes it: the coefficient for NSSCR falls to −0.343 (p=0.284) and that for mean TVSymp to −0.025 (p=0.659), with the partial $\eta^{2}$ for group falling from 0.224 to 0.042 and from 0.188 to 0.007, respectively. In the fully adjusted model, the TVSymp partial regression coefficient is close to zero (0.005). The variance inflation factor for the group term is 2.31 in the fully adjusted model, which is moderate rather than extreme, indicating that this attenuation is unlikely to be the artifact of an unstable fit. Therefore, we cannot separate an effect of AD from an effect of age on phasic electrodermal activity: the unadjusted group differences are large and reproducible, but they are statistically attributable to the age difference between the groups to an extent that this study cannot resolve.

Of the three stratified comparisons, the etiology-restricted analysis is the most informative, because it reduces the age difference substantially without discarding the disease contrast. Restricting the AD group to the six participants from the autosomal-dominant kindreds (mean age 50.5 years) brings the mean age difference against the control group down from 23.5 years to 6.4 years, giving a nearly age-matched comparison within the existing data, albeit in a small subgroup. In that comparison, neither feature differed significantly and both effect sizes fell by roughly two thirds: NSSCR was 1.85±0.40 in controls versus 1.59±1.05 in the six kindred patients (p=0.486, Cohen’s d=−0.37), and mean TVSymp was 1.00±0.11 versus 0.97±0.13 (p=0.614, d=−0.27). This result corroborates with the age-adjusted findings but remains non-confirmatory given the underpowered sample size of six AD patients.

The sex-restricted analysis compares the nine female controls with the 9 female AD participants (Fisher’s exact test for the sex imbalance across the full groups, p=0.024). Two features of this subgroup should be borne in mind. It has 80% power only for effects of d≥1.41, whereas the effects observed in the full sample were d=−1.07 and −0.96. Moreover, it does not hold age constant: the female AD participants are drawn disproportionately from the older sporadic subgroup, so the mean age difference within the female subgroup is approximately 32 years, larger than the 23.5 years in the full sample. In this comparison, NSSCR differed with p=0.0022 and d=−1.71 and mean TVSymp with p=0.080 and d=−0.88.

Excluding the six AD participants receiving cholinesterase inhibitor therapy, both differences were preserved and larger than in the full sample (NSSCR p=0.0003, d=−1.78; mean TVSymp p=0.0019, d=−1.46), suggesting the group difference in phasic activity is unlikely to be an artifact of cholinergic medication.

Additionally, we performed an analysis that excluded the single participant staged as MCI rather than dementia, which left both results essentially unchanged (NSSCR p=0.012, d=−1.06; mean TVSymp p=0.015, d=−1.02; against p=0.0083, d=−1.10 and p=0.0166, d=−0.99 with that participant included).

All three subgroup comparisons are underpowered relative to the primary analysis, and we report the effect sizes alongside the *p*-values so that a non-significant result in a small subgroup is not construed as evidence of no effect.

### 3.4. Classification Performance

Table 4 summarizes the classification performance of each model, with 95% confidence intervals. The highest point estimate of balanced accuracy, 0.78 with a 95% confidence interval of 0.60 to 0.93, was obtained from the random forest model trained with the features obtained during the MoCA.

The three MoCA models are not statistically distinguishable. Their balanced accuracy confidence intervals overlap almost completely, and the random forest’s advantage over the two linear models amounts to a single additional correctly classified participant out of 30 (McNemar’s exact test, p=1.00). The ordering also reverses depending on the metric: although the random forest attains the highest balanced accuracy, the support vector machine attains the highest AUROC, 0.785 [0.621, 0.949] against 0.745 [0.567, 0.923] for the random forest, and the three AUROC intervals too overlap almost entirely. Since the ranking reverses between metrics and margins remain well within confidence intervals, we do not claim that any one model outperforms the others.

**Figure 4:**
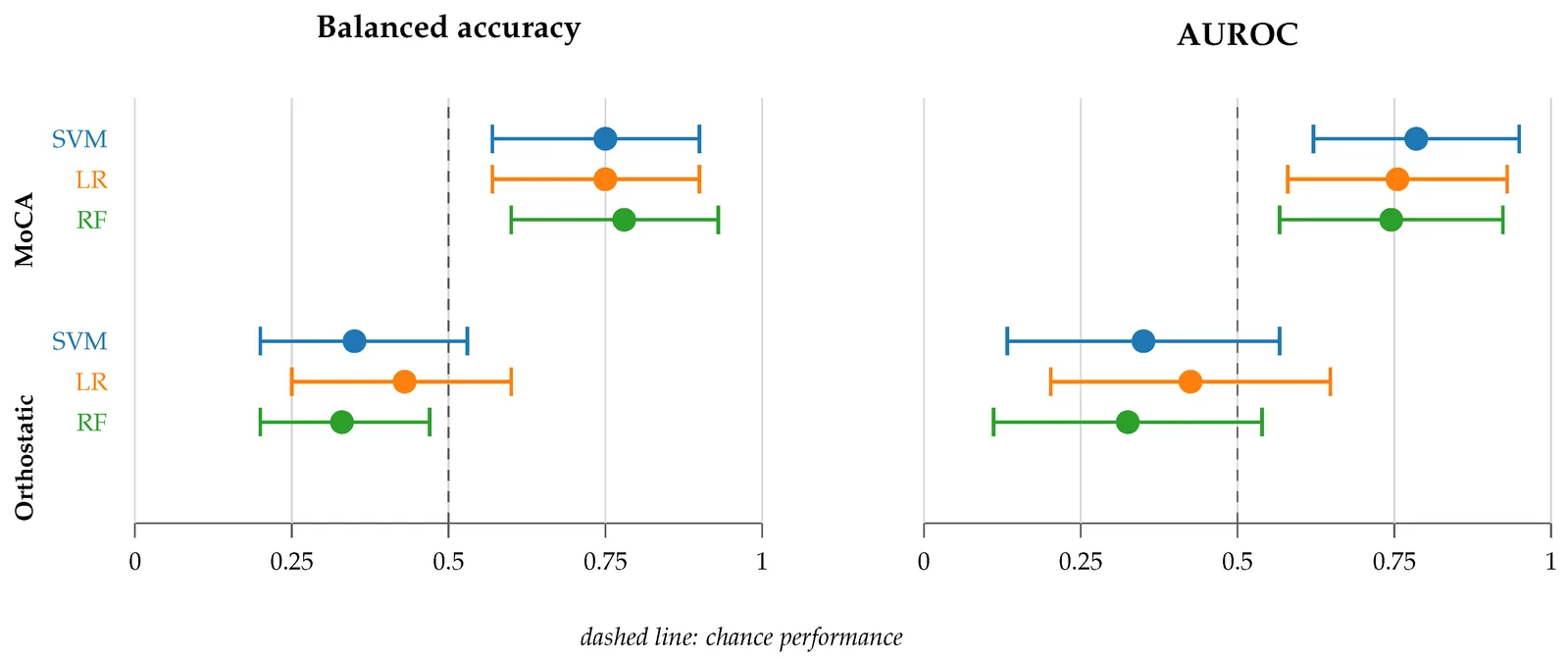
Comparison of the three classifiers with 95% confidence intervals, for both recording conditions. Points are the estimates reported in Table 4 and horizontal bars the corresponding intervals: a stratified subject-level bootstrap for balanced accuracy and the method of Hanley and McNeil for the AUROC. The intervals for the support vector machine, logistic regression and random forest overlap almost completely within each condition, and the ordering of the three models reverses between the two metrics, with the random forest attaining the highest balanced accuracy during the Montreal Cognitive Assessment and the support vector machine attaining the highest AUROC. McNemar’s exact test on the paired leave-one-subject-out predictions gave p=1.00 for every pairwise comparison. The three models are therefore not statistically distinguishable, and the contrast is between the two recording conditions rather than between model families. SVM: support vector machine; LR: logistic regression; RF: random forest.

Figure 4 shows the AUROC and balanced accuracy estimates and their intervals side by side for both the MoCA and the orthostatic test and makes the overlap immediately apparent. We report that the three models trained on MoCA-derived EDA features separate the groups appreciably better than chance, whereas none of the models trained on orthostatic test features do.

**Figure 5:**
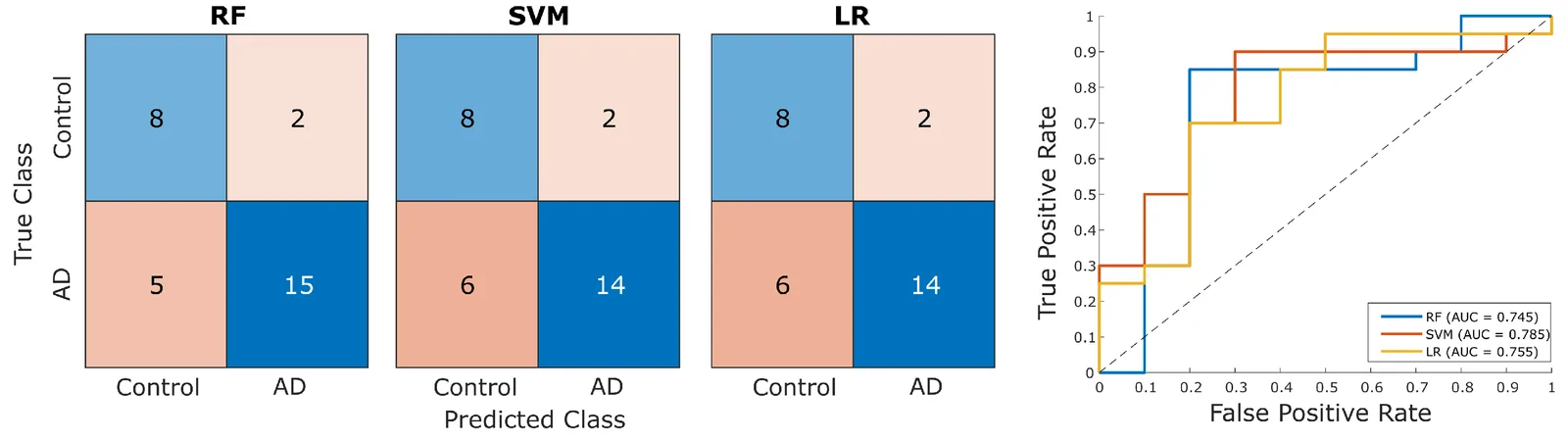
Classification results for the three models trained on EDA features recorded during the Montreal Cognitive Assessment. Left three panels: confusion matrices for the random forest (RF), support vector machine (SVM), and logistic regression (LR) models; cell values are numbers of participants, aggregated over the leave-one-subject-out folds. Right panel: receiver operating characteristic curves for the three models, with the corresponding areas under the curve. The dashed diagonal indicates chance performance.

For all three models trained on the MoCA features, the confusion matrices and the ROC curves are provided in Figure 5. Permutation importance analysis based on balanced accuracy further revealed the feature contributions for these models. For the random forest model, NSSCR exhibited the largest decrease in performance when permuted (mean drop = 0.172), followed by mean TVSymp (0.158) and EDASympn (0.004), alongside a small performance *increase* when SCL was permuted (mean drop = −0.016), suggesting that this feature played no positive role in model performance. For the support vector machine and logistic regression models, NSSCR again exhibited the largest decrease in performance (0.248 and 0.239, respectively), followed by mean TVSymp (0.064 and 0.076), EDASympn (0.028 and 0.037), and SCL (0.023 and 0.025). Given the limited sample size, we focus on the rank ordering rather than the exact magnitude of performance changes. We find that the two phasic features (NSSCR and mean TVSymp) rank above the two remaining features in all three model families.

## 4. Discussion

To determine whether EDA can serve as an inexpensive, non-invasive tool for AD screening, we carried out a pilot study that provides preliminary evidence that EDA features can distinguish between control and AD populations. During the MoCA, NSSCR and mean TVSymp, which reflect phasic EDA components, were markedly elevated in the control group relative to the AD group, with large effect sizes (Cohen’s d=−1.07, p=0.0083 and d=−0.96, p=0.0166, respectively). Although only NSSCR remained significant after Bonferroni correction, the comparable effect size and consistent directionality of both features suggest that phasic EDA measures are sensitive to differential sympathetic arousal between AD and control groups.

Consequently, machine learning models trained on MoCA-derived EDA features achieved moderate classification performance, with balanced accuracies of 0.75 to 0.78 and AUROCs of 0.75 to 0.79 across the three model families. Permutation importance analysis across all three MoCA classification models consistently identified NSSCR as the leading contributor to model performance, followed by mean TVSymp. The fact that this ranking held across three structurally distinct model families, a tree-based ensemble, a linear kernel method, and a linear classifier, strengthens confidence that the association between phasic EDA and AD status reflects a genuine physiological signal rather than an artifact of any single modeling approach. The three models did not differ significantly from one another on any metric, with relative rankings reversing depending on the evaluation measure. These results do not suggest a best-performing model and instead indicate that the predictive signal in these features is model-agnostic. In contrast, classifiers trained on orthostatic test features demonstrated poor performance, with the best balanced accuracy reaching only 0.43 (95% CI 0.25 to 0.60) using logistic regression, consistent with the absence of significant group-level differences in that condition.

The physiological differences observed during the MoCA may be related to the cognitive and performance-related stress elicited by the test. Previous work has shown that cognitively demanding tasks increase EDA in healthy individuals, including elevations in SCL [60], NSSCR [61], and stimulus-locked skin conductance responses more broadly, which are understood to index task engagement and effortful cognitive processing [62]. We therefore hypothesized that AD subjects would exhibit a blunted EDA response during cognitive challenge as a result of autonomic dysfunction, and our findings are consistent with this hypothesis.

Notably, the group differences we observed were confined to phasic EDA measures, while tonic activity (SCL) did not differ significantly between groups. This dissociation is compatible with the distinct physiological generators of phasic and tonic EDA: phasic responses arise from rapid, stimulus-driven bursts of sympathetic sudomotor activity, whereas tonic SCL reflects slower-changing baseline arousal and is also influenced by non-neural factors such as skin hydration and ambient temperature [46].

One candidate neurobiological account of this pattern involves the locus coeruleus (LC), the brain’s principal source of noradrenergic output and a key driver of phasic sympathetic responses to salient or effortful stimuli [63]. LC neuronal loss is among the earliest detectable neuropathological changes in AD and results in reduced noradrenergic drive to downstream sympathetic effector systems, including the sudomotor pathways that generate skin conductance responses [63]. However, this account faces two challenges. First, LC degeneration is not specific to AD: comparable noradrenergic cell loss and reduced neuromelanin-based LC contrast are observed in Parkinson’s disease, dementia with Lewy bodies, and other neurodegenerative conditions [64]. Second, and more consequentially for the present data, LC integrity declines with normal aging, so in a cohort whose groups differ by approximately 23.5 years in mean age, an LC-mediated explanation is empirically indistinguishable from age-related attrition of noradrenergic innervation of the sudomotor system [64,65]. We therefore do not advance LC degeneration as an explanation of our findings. We retain it as one of several hypotheses that the present design cannot arbitrate, and note that it becomes testable only in a study that either matches groups on age or measures LC integrity directly, for instance by pairing EDA with neuromelanin-sensitive MRI or cerebrospinal fluid noradrenaline measures, in which case LC integrity rather than diagnostic group would be the appropriate predictor.

### 4.1. The Orthostatic Test

We expected the orthostatic test to reveal physiological differences related to orthostatic intolerance in our AD cohort, 45% of whom reported orthostatic symptoms on the COMPASS-31 questionnaire [42]. However, we found no significant between-group differences in any EDA feature, and, as reported in Section 3.1, the blood-pressure criterion applied during the test itself identified only one participant in each group despite that documented prevalence. Because a single post-standing reading frequently misses the blood-pressure nadir, we attribute both negative findings primarily to the brevity of the one-minute standing period. It is likely that this one minute of standing is, on physiological grounds, inadequate for the features we computed. NSSCR is a rate estimated from a count of discrete events, and at the observed response rates of roughly 1.3 responses per minute, a 60 s window yields on the order of one or two events per participant, so the estimator may be dominated by counting noise. EDASympn and TVSymp are spectral and time–frequency quantities defined over the 0.01 to 0.24 Hz band, whose lowest component has a period of 100 s, longer than the entire standing epoch, so the low-frequency end of the band is not resolvable in a 60 s record. Prior work on comparable slow autonomic dynamics has recommended standing and sitting periods of at least four minutes each [24]. The negative result for the orthostatic test should therefore be read as a limitation of the recording window rather than as evidence that orthostatic autonomic responses are preserved in AD. Future studies should extend the standing phase to at least four minutes, subject to participant tolerance and fall-risk precautions, and may also benefit from jointly analyzing the blood pressure measurements collected alongside EDA in this protocol, which were not statistically evaluated in the present study.

### 4.2. Toward Remote Screening

Although both the MoCA and the orthostatic test were administered in a clinical setting in this study, these protocols were selected because they are well suited for extension to telehealth and home-based assessments. Home-based orthostatic testing has been shown to be reliable [66], and large-scale studies have similarly demonstrated the validity of remotely administered MoCA assessments [67]. Moreover, EDA is amenable to continuous, unobtrusive monitoring via wearable devices, with demonstrated success in tracking stress and behavioral changes [30,31,32], as well as recent technological innovations further enhancing its suitability for wearable applications [22]. Taken together, these considerations suggest it may be feasible to develop multimodal models integrating orthostatic test responses, MoCA performance, and EDA-derived features for remote screening of AD risk. We note, however, that the ambient conditions that a clinic can standardize, namely temperature, humidity, acoustic environment, and posture, are precisely those that are hardest to control at home, and that this represents a substantive obstacle to translation rather than an implementation detail [45,47].

### 4.3. Limitations

Given that this was a pilot study intended to motivate future investigations, its findings should be regarded as preliminary and hypothesis-generating, and several limitations warrant discussion.

Age. A major limitation is the substantial age difference between groups, approximately 23.5 years (Table 1). Aging is independently associated with changes in autonomic function, including sudomotor activity [65], and EDA measures in particular have been shown to differ systematically between younger and older adults, with reduced electrodermal reactivity in older participants [51]. The observed differences therefore cannot be attributed exclusively to AD pathology, and age-related autonomic decline remains a plausible, and in this cohort largely inseparable, alternative explanation. Our covariate-adjusted analyses in Section 2.5.1 are limited by strong collinearity between group and age, which statistical adjustment cannot fully resolve. Analyzing the etiology-restricted subgroup in Section 3.3 partially addresses this age disparity, though the small sample size limits statistical power. Future studies must employ age-matched cohorts.

Sex. The two groups are also imbalanced in sex composition: the control group was 90% female, with 9 female and 1 male participant, whereas the AD group was 45% female, with 9 female and 11 male participants. This may be a physiological confounder for our measurements, since eccrine sweat gland density, sudomotor output, and both tonic and phasic electrodermal parameters have been reported to differ between men and women [46,52]. As with age, sex is thus entangled with group membership; this unintentional imbalance arose from the clinic convenience sampling described in Section 2.1.

Restricting the analysis to women (Section 3.3) eliminates the sex confound, but at the cost of statistical power (80% power only for effects of d≥1.41) and a wider age gap; the female AD participants were drawn disproportionately from the older sporadic subgroup and on average 14 years older than the male participants (p=0.022).

Generalizability across AD etiologies. Our AD group comprised both participants from Colombian kindreds affected by autosomal-dominant AD and patients with sporadic late-onset AD, and these two forms of the disease differ in ways that bear directly on our measurements. Autosomal-dominant AD has a far earlier and more predictable age at symptom onset, a more rapid and stereotyped progression, and a distinct topography and tempo of neurodegeneration relative to sporadic disease [37,38,68]. Because carriers develop AD pathology at ages at which the confounding burden of vascular disease, polypharmacy, and other age-related comorbidity is comparatively low, the pathophysiological signal may be cleaner in this population than in a sporadic cohort, but for the same reason it may also be quantitatively different. There is at present no evidence establishing that patterns of autonomic dysfunction, or of LC and noradrenergic degeneration, are equivalent in monogenic mutation carriers and in typical late-onset AD, and it is entirely possible that they are not. Our study is not powered to test that equivalence, and the etiology-restricted comparison reported in Section 3.3 is descriptive rather than confirmatory. Sporadic late-onset AD accounts for the overwhelming majority of clinical cases and is the population for which an EDA-based screening tool would ultimately be intended, so establishing whether the effect we report holds in a purely sporadic cohort, and whether it is quantitatively comparable across etiologies, is a necessary step before any translational claim can be made.

Medication status. AD participants may receive pharmacotherapy that acts on the very system EDA measures. Eccrine sweat glands are innervated by sympathetic fibers that are cholinergic and act on muscarinic receptors, so cholinesterase inhibitors such as donepezil and rivastigmine have a mechanistically direct route to influencing skin conductance, and their systemic autonomic effects are known to be complex and to vary with acute versus chronic administration [41].

The sensitivity analysis excluding cholinesterase inhibitor treated participants (Section 3.3), although underpowered, reveals consistent effect sizes and directionality with the original results. Because cholinergic augmentation enhances sudomotor output, any treatment bias would be expected to inflate phasic EDA, potentially making our observed reductions conservative.

A similar limitation applies to the antihypertensive, beta-blocker, antidepressant, and antipsychotic agents recorded in Table 1. Since these medications were present almost exclusively in the AD group, their potential confounding effects cannot be adequately disentangled from disease status in a sample of this size.

Sample size and model reliability. The overall sample of 30 participants, unequally allocated, is small in absolute terms and small in particular for the machine learning component. Classification performance estimated from 30 subjects carries very wide uncertainty, with the confidence intervals in Table 4 spanning roughly 30 percentage points, and cross-validated performance estimates in small samples are known to be both highly variable and optimistically biased relative to independent validation [59]. The models reported here are a demonstration that the features carry group-discriminative information, and their generalizability and reproducibility remain to be established in an independent cohort. For the same reason, the permutation importance estimates are unstable: model-agnostic importance measures are sensitive to sampling variability and to correlations among features, and are known to be unreliable at these sample sizes [58]. We interpret only the consistent ordering of features across three model families, and not the magnitude of any individual importance value. A related methodological limitation concerns the use of SMOTE to address the class imbalance in our classification pipeline. Because our control group contained only 10 subjects, the *k*-nearest-neighbor interpolation underlying SMOTE had a limited pool of real samples from which to generate synthetic minority-class examples, which may reduce the reliability of the synthetic data relative to applications with larger minority classes.

Measurement conditions. EDA measurements are sensitive to motion artifacts, speech, ambient temperature and humidity, background noise, and variations in emotional state [45,46,47]. In this study, motion artifacts were mitigated by instruction, hand positioning, median filtering, and automated quality assessment with EDAQA [49]; ambient temperature and humidity were actively controlled; and variability in emotional state was partially addressed through consistent environmental conditions, though it was not directly measured.

Three residual issues deserve emphasis. First, the MoCA requires participants to speak, and speech elicits skin conductance responses; this paradigm applies equally to both groups. However, the direction of any speech-related bias would be expected to raise the response count of the group that speaks or is spoken to more, and AD participants, who require more prompting and more examiner interaction, would if anything be biased towards higher NSSCR, whereas we observed lower NSSCR in that group. Nevertheless, we cannot exclude a contribution from differences in verbal output between groups. Second, although the recording room was temperature- and humidity-controlled, these variables were not logged during individual sessions, so we can report that conditions were nominally constant across participants but cannot demonstrate it from measurements. Third, a single reusable dry electrode pair was used for all participants. Dry contacts have a higher and more variable electrode-to-skin impedance than pre-gelled wet electrodes, and although a fixed five-minute stabilization interval preceded every recording and the pair was cleaned between participants, we did not measure contact impedance and therefore cannot verify that the interface was equivalent across sessions.

Statistical power. To inform future study design and contextualize the MoCA findings, a post hoc power analysis was conducted [69]. Power calculations were performed analytically using the *statsmodels 0.14.6* library in Python 3.14 [50]. Under the present unequal allocation of 10 controls and 20 AD subjects, the achieved power was approximately 0.54 for NSSCR and 0.44 for mean TVSymp, indicating that the study was underpowered even for the largest observed effects. Based on the observed effect sizes, an age-matched replication preserving the current allocation ratio would require approximately 48 to 59 participants to achieve 80% power, while a more conservative design assuming moderate effects (d=0.6) would require on the order of 140 participants. We note that power calculations based on observed effect sizes are themselves biased upward when computed from a small, significant sample, so these figures should be treated as a lower bound on the required sample size [39].

### 4.4. Future Work

The design of a confirmatory study follows directly from the limitations above: age- and sex-matched groups; a sporadic late-onset AD cohort large enough to be analyzed in its own right; documented medication status with pre-specified sensitivity analyses; an extended orthostatic protocol of at least four minutes per posture; and a sample size informed by the power analysis reported above.

Another important direction is to extend the study to individuals with mild cognitive impairment (MCI), as this stage represents the critical window for early intervention and represents the diagnostic challenge a clinical screening tool would encounter in practice. The comparison we report here, established dementia versus cognitively healthy adults, is the easiest version of the problem, and good performance on it does not imply performance on the clinically relevant contrast. An investigation with MCI subjects would help clarify whether the co-occurrence of autonomic dysfunction and early-stage AD pathology, prior to a formal AD diagnosis, is detectable through EDA features. Demonstrating sensitivity at the MCI stage would also provide a foundation for subsequent studies aimed at real-world deployment, potentially enabling remote AD risk screening and longitudinal remote monitoring of disease progression.

Extending the single-channel analysis reported here toward the multimodal, clinically deployable framework outlined above will also require attention to the methodological questions that now dominate clinically oriented machine learning: how heterogeneous modalities are combined, how a model’s behavior is made interpretable to the clinician who must act on it, and how performance is sustained across sites, devices and populations. Recent work on multimodal fusion for diagnosis in other clinical domains illustrates architectures that address the first two of these concerns while anchoring validation in clinically confirmed outcomes [70]. Our own results speak directly to the third: three structurally different classifiers performed indistinguishably, and the cross-validated intervals were wide, so external validation in an independent cohort, rather than added model complexity, is the step most likely to establish whether an EDA-based screening tool generalizes.

## 5. Conclusions

This pilot study involved collecting EDA signals from 10 control and 20 AD subjects while they underwent an orthostatic test and a MoCA, with the objective of evaluating whether EDA-derived features can act as biomarkers for AD. During the MoCA, controls exhibited significantly elevated phasic EDA compared to AD subjects (NSSCR: p=0.0083; mean TVSymp: p=0.0166), and classifiers trained on these features separated the groups with balanced accuracies of 0.75 to 0.78, with no significant differences among model families. No differences were detected during the 60-s orthostatic challenge, a window we argue is too short to resolve the relevant autonomic dynamics. These findings provide preliminary evidence that EDA may have utility as a low-cost, non-invasive tool for AD assessment, but they are constrained by the small and unequally allocated sample, and by group differences in age and sex that cannot be separated from disease status in this design. They should be interpreted as hypothesis-generating rather than as evidence of diagnostic utility. Future work should seek to validate these results in larger cohorts matched in age and sex, in sporadic late-onset AD, and including subjects with MCI.

## Figures and Tables

**Table 1:** Demographic and clinical characteristics of the study groups. Continuous variables are reported as mean ± standard deviation; categorical variables as counts. The final column reports between-group comparisons, using independent-samples *t*-tests for continuous variables and Fisher’s exact test for categorical variables. ChEI: cholinesterase inhibitor; FAST: Functional Assessment Staging Tool; MoCA: Montreal Cognitive Assessment; OH: orthostatic hypotension, defined as a fall of ≥20 mmHg systolic or ≥10 mmHg diastolic between the seated and standing measurements of the orthostatic test. COMPASS-31 is the Composite Autonomic Symptom Score, a self-report instrument administered earlier in the same session.

Characteristic	Control (n=10)	AD (n=20)	*p*
Age (years)	44.1 ± 7.28	67.6 ± 16.22	<0.001
Sex (female/male)	9/1	9/11	0.024
Autosomal-dominant kindred/sporadic	8/2	6/14	0.019
MoCA score (0 to 30)	24.6 ± 3.27	9.2 ± 6.20	<0.001
MoCA administration time (min)	8.9 ± 2.38	16.1 ± 9.28	0.003
FAST stage (median [range])	1 [1 to 1]	4 [1 to 6]	n/a
Hypertension (*n*)	0	9	0.013
Diabetes mellitus (*n*)	0	0	1.000
Cholinesterase inhibitor use (*n*)	0	6	0.074
Antihypertensive or beta-blocker (*n*)	0	7	0.064
Antidepressant or antipsychotic (*n*)	1	9	0.101
Orthostatic symptoms on COMPASS-31 (*n*)	4 (40%)	9 (45%)	1.000
Met OH criterion during test (*n*)	1/10	0/20	0.333
Staged as MCI rather than dementia (*n*)	n/a	1	n/a

**Table 2:** Between-group comparison of electrodermal activity features during the orthostatic test and the Montreal Cognitive Assessment. Values are mean ± standard deviation, followed by the 95% confidence interval of the mean in brackets. For each test, the final two rows report Cohen’s *d* for the AD-versus-control contrast, where negative values indicate lower values in the AD group, and the corresponding *p*-value; for the orthostatic test, the contrast is computed on the standing-minus-sitting change. *p*-values are from unpaired *t*-tests, or Wilcoxon rank-sum tests where normality was violated. The Bonferroni-corrected significance threshold is p<0.0125. SCL: skin conductance level; NSSCR: number of skin conductance responses; EDASympn: normalized time-invariant EDA sympathetic index; TVSymp: time-variant sympathetic tone; n.u.: normalized units. * p<0.05; ** p<0.0125, significant after Bonferroni correction.

Test	Group	SCL (µS)	NSSCR (Count/min)	EDASympn (n.u.)	Mean TVSymp (n.u.)
**Orthostatic**	AD (Sit)	1.38 ± 1.40 [0.77, 1.99]	1.38 ± 0.68 [1.08, 1.68]	0.001 ± 0.001 [0.000, 0.002]	0.82 ± 0.20 [0.73, 0.91]
	Control (Sit)	1.33 ± 0.99 [0.71, 1.94]	1.29 ± 0.68 [0.87, 1.71]	0.001 ± 0.002 [0.000, 0.003]	0.67 ± 0.22 [0.53, 0.81]
	AD (Stand)	1.42 ± 1.34 [0.84, 2.01]	1.35 ± 1.51 [0.69, 2.01]	0.003 ± 0.009 [0.001, 0.011]	0.95 ± 0.54 [0.72, 1.19]
	Control (Stand)	1.37 ± 1.08 [0.70, 2.04]	1.28 ± 1.44 [0.39, 2.17]	0.001 ± 0.001 [0.000, 0.002]	0.94 ± 0.50 [0.63, 1.25]
	Cohen’s *d* (Stand minus Sit)	0.02	−0.02	0.26	−0.20
	*p*	0.9654	0.9641	0.7749	0.6029
**MoCA**	AD	1.31 ± 1.38 [0.67, 1.96]	1.13 ± 0.75 [0.78, 1.48]	0.001 ± 0.002 [0.001, 0.003]	0.88 ± 0.13 [0.82, 0.94]
	Control	1.83 ± 1.00 [1.11, 2.54]	1.85 ± 0.40 [1.56, 2.14]	0.001 ± 0.000 [0.000, 0.001]	1.00 ± 0.11 [0.92, 1.08]
	Cohen’s *d*	−0.40	−1.07	0.35	−0.96
	*p*	0.3028	0.0083 **	0.4953	0.0166 *

**Table 3:** Effect of the AD-versus-control contrast on the two phasic MoCA features, unadjusted and after adjustment for age and sex in a multivariable linear model. $\beta_{1}$ is the partial regression coefficient for group, with AD coded 1 and control 0. VIF is the variance inflation factor for the group term, which quantifies how far the group effect is entangled with the covariates. Given that group and age are near-collinear in this cohort, these models are reported as sensitivity analyses rather than as de-confounded effect estimates.

Feature	Model	$\beta_{1}$ (95% CI)	*p*	Partial $\eta^{2}$/VIF
NSSCR	Unadjusted	−0.720 [−1.239, −0.201]	0.0083	0.224/n/a
	+age	−0.343 [−0.986, +0.301]	0.284	0.042/1.67
	+sex	−0.858 [−1.432, −0.284]	0.0049	0.258/1.23
	+age + sex	−0.456 [−1.223, +0.311]	0.233	0.054/2.31
Mean TVSymp	Unadjusted	−0.121 [−0.219, −0.024]	0.0166	0.188/n/a
	+age	−0.025 [−0.138, +0.089]	0.659	0.007/1.67
	+sex	−0.123 [−0.233, −0.013]	0.030	0.163/1.23
	+age + sex	+0.005 [−0.129, +0.140]	0.937	0.000/2.31

**Table 4:** Performance of the three classification models trained on EDA features, evaluated by leave-one-subject-out cross-validation over all 30 participants. Values are point estimates with 95% confidence intervals: Wilson score intervals for sensitivity and specificity, a stratified subject-level bootstrap interval over 10,000 resamples for balanced accuracy, and intervals computed by the method of Hanley and McNeil for the AUROC. Balanced accuracy is the mean of sensitivity and specificity. For the orthostatic test models, whose receiver operating characteristic curves were not retained, the area reported is the trapezoidal area through the single operating point defined by the tabulated sensitivity and specificity, which equals the balanced accuracy and is a lower bound on the area obtainable from the continuous scores. Confidence intervals overlap extensively across models within each test, and pairwise comparisons were not statistically significant, as reported in the text. SVM: support vector machine; LR: logistic regression; RF: random forest; AUROC: area under the receiver operating characteristic curve.

Test	Model	Sensitivity	Specificity	Balanced Accuracy	AUROC
Orthostatic	SVM	0.50 [0.30, 0.70]	0.20 [0.06, 0.51]	0.35 [0.20, 0.53]	0.350 [0.133, 0.567]
	LR	0.55 [0.34, 0.74]	0.30 [0.11, 0.60]	0.43 [0.25, 0.60]	0.425 [0.202, 0.648]
	RF	0.55 [0.34, 0.74]	0.10 [0.02, 0.40]	0.33 [0.20, 0.47]	0.325 [0.111, 0.539]
MoCA	SVM	0.70 [0.48, 0.85]	0.80 [0.49, 0.94]	0.75 [0.57, 0.90]	0.785 [0.621, 0.949]
	LR	0.70 [0.48, 0.85]	0.80 [0.49, 0.94]	0.75 [0.57, 0.90]	0.755 [0.580, 0.930]
	RF	0.75 [0.53, 0.89]	0.80 [0.49, 0.94]	0.78 [0.60, 0.93]	0.745 [0.567, 0.923]

## Data Availability

The data presented in this study were collected previously by the Grupo de Neurociencias de Antioquia (GNA), Universidad de Antioquia, and are not publicly available due to institutional and participant privacy restrictions. Data may be made available by GNA upon reasonable request, subject to applicable data sharing agreements and ethical approval requirements.

## Abbreviations

The following abbreviations are used in this manuscript:

AD	Alzheimer’s disease
AUROC	Area under the receiver operating characteristic curve
ChEI	Cholinesterase inhibitor
CI	Confidence interval
EDA	Electrodermal activity
EDASympn	Time-invariant sympathetic index (normalized EDA sympathetic index)
FAST	Functional Assessment Staging Tool
fMRI	Functional Magnetic Resonance Imaging
FIR	Finite Impulse Response
GNA	Grupo de Neurociencias de Antioquia
HRV	Heart rate variability
LC	Locus Coeruleus
LR	Logistic regression
MCI	Mild cognitive impairment
MoCA	Montreal Cognitive Assessment
MRI	Magnetic resonance imaging
NSSCR	Number of skin conductance responses
OH	Orthostatic hypotension
PET	Positron emission tomography
RF	Random forest
ROC	Receiver operating characteristic
SCL	Skin conductance level
SD	Standard deviation
SMOTE	Synthetic Minority Oversampling Technique
SVM	Support vector machine
TVSymp	Time-variant sympathetic tone
VFCDM	Variable Frequency Complex Demodulation
VIF	Variance inflation factor
